# Estrogen Receptors in Polycystic Ovary Syndrome

**DOI:** 10.3390/cells10020459

**Published:** 2021-02-21

**Authors:** Xue-Ling Xu, Shou-Long Deng, Zheng-Xing Lian, Kun Yu

**Affiliations:** 1 College of Animal Science and Technology, China Agricultural University, Beijing 100193, China; xusnowling@163.com; 2 Institute of Laboratory Animal Sciences, Chinese Academy of Medical Sciences, Ministry of Health, Beijing 100021, China; dengsl@big.ac.cn; 3 CAS Key Laboratory of Genome Sciences and Information, Beijing Institute of Genomics, Chinese Academy of Sciences, Beijing 100101, China

**Keywords:** estrogen, estrogen receptor, ovary, polycystic ovary syndrome

## Abstract

Female infertility is mainly caused by ovulation disorders, which affect female reproduction and pregnancy worldwide, with polycystic ovary syndrome (PCOS) being the most prevalent of these. PCOS is a frequent endocrine disease that is associated with abnormal function of the female sex hormone estrogen and estrogen receptors (ERs). Estrogens mediate genomic effects through ERα and ERβ in target tissues. The G-protein-coupled estrogen receptor (GPER) has recently been described as mediating the non-genomic signaling of estrogen. Changes in estrogen receptor signaling pathways affect cellular activities, such as ovulation; cell cycle phase; and cell proliferation, migration, and invasion. Over the years, some selective estrogen receptor modulators (SERMs) have made substantial strides in clinical applications for subfertility with PCOS, such as tamoxifen and clomiphene, however the role of ER in PCOS still needs to be understood. This article focuses on the recent progress in PCOS caused by the abnormal expression of estrogen and ERs in the ovaries and uterus, and the clinical application of related targeted small-molecule drugs.

## 1. Introduction

Polycystic ovary syndrome (PCOS) is a type of general disease in women that is associated with a variety of reproductive and metabolic disorders [1]. The symptom is characterized by arrested folliculogenesis, hyperandrogenism, and polycystic ovaries [2]. Women suffering from PCOS account for a large proportion of the world population, with the prevalence estimated at between 5% and 10% [3]. Since the National Institutes of Health established a more standardized diagnosis standard in 1990, and with the continuous revision of this standard, the prevalence of PCOS is now expected to be close to 18% in the United States [4]. People with PCOS have more adverse reproductive risks, such as increased incidence of implantation failure, recurrent abortion, spontaneous abortion, premature birth, and endometrial carcinoma [5,6]. For example, women with PCOS were shown to have a 2.7-fold increased risk of endometrial cancer [7]. In addition to reproductive abnormalities, PCOS is also closely related to large quantities of metabolic disorders, for instance increased risk of hyperlipidemia, diabetes mellitus type II (T2DM), and hypertension, as well as hepatic steatosis, glucose intolerance, and insulin resistance [8,9].

Normal ovulation is the result of synergy between follicle-stimulating hormone (FSH) and luteinizing hormone (LH). LH stimulates the theca cells of the ovarian follicle, leading to androgen synthesis. Some of these are bound to sex-hormone-binding globulin (SHBG), and some androgens spread to nearby granulosa cells (GCs), where they are converted to estrogen under the stimulation of FSH. This causes an increase in the level of estrogen hormones, which in turn generates positive feedback in the form of LH production, causing a surge in LH and triggering ovulation [10]. The proliferation and differentiation of theca cells and granulosa cells are regulated by locally produced growth factors. Therefore, ovarian follicular development is tightly regulated by many hormones and other growth factors [11]. After ovulation, the corpus luteum is formed, which secretes the steroid hormones progesterone and estrogen, and then makes the endometrium more receptive to implantation [12].

Research on women with PCOS indicates elevated production of LH and free testosterone. Under the influence of endocrine-disrupting chemicals (phytoestrogens or synthetic estrogenic compounds), hormone secretion is also further affected, resulting in the PCOS disease [13]. As its name implies, the disease is a state of chronic anovulation that involves many ovarian cysts, characterized by an increased number of immature cystic follicles [14]. The ovaries are the primary site of estrogen synthesis, mainly producing 17β-estradiol (E2), which exerts effects on target organs and cells via multiple estrogen receptors (ERs), ERα, ERβ, and the G-protein-coupled estrogen receptor (GPER, also known as GPR30) to maintain various stages of normal development in the human ovaries and uterus. In this review, we will discuss the latest advances in the understanding of how ER mediates the role of estrogen in polycystic ovary syndrome, with an outlook for possible clinical treatments.

## 2. The Characteristics of Estrogen Receptors

### 2.1. Estrogen Receptors: Expression, Structure

Estrogen mediates most biological effects at the gene level through estrogen receptors; maintains normal reproductive function; and plays key roles in the musculoskeletal, cardiovascular, immune, and central nervous systems [15]. As members of the nuclear receptor family of transcription factors, estrogen receptors occur not only in the nucleus, but also in the cytoplasm and mitochondria of cells. In 1958, Elwood Jensen discovered the first ER (known as ERα). It was subsequently demonstrated that conjugated estrogen receptors could migrate to the nucleus, thereby stimulating gene transcription [16,17]. Similarly, a second ER gene, ERβ, was discovered by Jan-Ake Gustafsson in 1996 [18]. They discovered that ERβ was significantly highly expressed in the prostate and ovaries of rats by in situ hybridization, and that it is highly homologous to ERα. According to the current level of knowledge, the tissue distribution of the ER types is different. ERα is mainly expressed in the uterus, with a small amount expressed in the skin, ovaries, testis, and gut, whereas the expression of ERβ is found in the ovaries, prostate, colon, kidneys, cardiovascular system, and central nervous system (CNS) [19].

**Figure 1:**
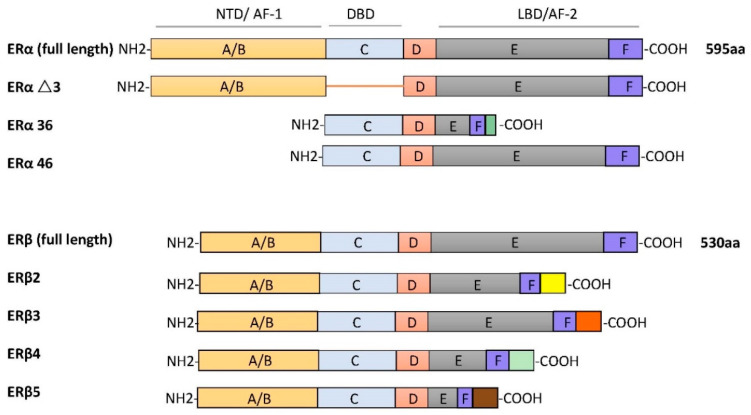
The schematic structures of estrogen receptor (ER) isoforms. Structural domains of ERα and ERβ are labeled A–F. Different functional domains are highlighted as follows: N- terminal (NTD/AF-1) in orange, DNA binding domain (DBD, C domain) in blue, the hinge (D domain) in red, and ligand-dependent transactivation function 2 (LBD/AF-2) in gray/dark blue.

ERα and ERβ are different receptors encoded by *Esr1* and *Esr2* on different chromosomes. ERα and ERβ are respectively found on human chromosome 6 and chromosome 14. The ERα protein is composed of 595 amino acids, with approximate molecular weight of 67 kDa. The full-length size of ERβ is 530 amino acids, with an approximate molecular weight of 59 kDa. Due to selective splicing of transcripts, they may occur in multiple isoforms. There are three ERα isoforms that have been identified. ERαΔ3 lacks exon 3, which encodes part of the DNA-binding domain. ERα36 lacks transcriptional activation domains (AF-1 and AF-2), but retains the DNA-binding domain and partial dimerization and ligand-binding domains [20]. ERα46 is produced by the selective splicing of an ERα gene product, which causes the start codon of exon 2 to initiate protein translation [21]. Four ERβ isoforms, ERβ2, ERβ3, ERβ4, and ERβ5, have been described [22]. All ERβ variants have a new C-terminus and do not bind to estrogen ligands that have been studied (as shown in Figure 1).

The structures of ERα and ERβ are composed of different functional domains. The main functional domains are called A/B, C, D, and E/F, which also have high sequence homology. The A/B region is the amino terminal domain (NTD), the C region corresponds to the DNA-binding domain (DBD), and the D domain is a hinge region connecting the C and E domains, capable of binding to chaperones. The carboxy terminal region contains the E/F region, also known as the ligand-binding domain (LBD) [23]. In silico analysis shows that two types of ERs have a common structural domain, namely the N terminal, DBD, and the C terminal, LBD [24]. These two receptors belong to the nuclear superfamily and function principally to regulate all kinds of cellular processes, such as proliferation, survival, differentiation, and apoptosis.

Another estrogen receptor, originally identified from human and rat tissues, is named orphan GPR30 [25,26]. Subsequent studies showed that GPR30 can specifically bind to estrogen and is a typical estrogen membrane receptor [27,28]. In 2007, GPR30 was officially named GPER, which has been extensively studied for its role in mediating a rapid response to estrogen, as well as overall physiological and pathological processes in human and animal models [29]. The gene encoding the membrane receptor GPER is located in chromosome 7. As a typical G-protein-coupled receptor, its structure consists of seven trans-membrane α-helical regions, four extracellular segments, and four cytosolic segments [30]. This receptor has a lower estrogen-binding affinity than other estrogen receptors. However, GPER is responsible for the rapid estrogen-mediated activation of ERK1/2 [31]. GPER is widely found in numerous human tissues, such as the reproductive system, prostate, ovaries, and placenta, as well as in the heart, liver, lungs, adipose tissue, and blood vessels [32].

### 2.2. Estrogen Receptor Ligands

Natural endogenous estrogens are mostly produced in the ovaries, corpus luteum, and placenta, predominantly 17β-estradiol, which is the main ligand of ERs. Various natural and man-made chemicals also have estrogenic activity. Phytoestrogens are plant-derived compounds with structural similarity to 17β-oestradiol, including isoflavones, prenylflavonoids, coumestans, and lignans [33,34]. Synthetic estrogenic compounds (also known as environmental estrogens) include pesticides, dioxins, phthalates, bisphenol A, and diethylstilbestrol [35,36]. They are often widely dispersed in the environment and result in developmental and reproductive abnormalities in humans.

It is believed that most phytoestrogens and synthetic estrogenic compounds exert their physiological effects by regulating ERα and ERβ [37]. Many of these compounds can also activate GPER, including the soy isoflavone genistein, nonylphenol, and bisphenol A [38].

**Figure 2:**
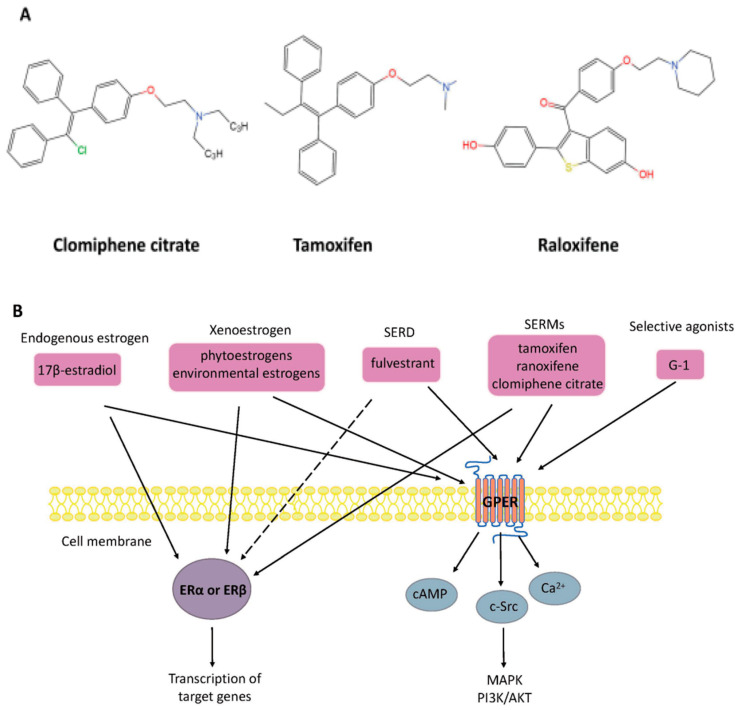
(**A**) Chemical structure of selective estrogen receptor modulators clomiphene citrate, tamoxifen, and raloxifene. (**B**) Nongenomic and genomic estrogen signal transduction pathways. Estrogen receptor ligands include 17β-estradiol and other compounds. Dashed lines indicate tissue-specific inhibition. SERD, selective estrogen receptor downregulator; SERM, selective estrogen receptor modulators; G-1, 1-[4-(6-bromobenzo[1,3] dioxol-5yl)-3a,4,5,9b-tetrahydro-3H-cyclopenta[c]quinolin-8-yl]-ethanone; ER, estrogen receptor; GPER, G-protein-coupled estrogen receptor; Ca2+, calcium ions; cAMP, cyclic adenosine monophosphate; MAPK, mitogen-activated protein kinase; PI3K/AKT, phosphatidylinositol 3-kinase/protein kinase B.

Other compounds are also widely used in clinical and therapeutic applications. For example, selective estrogen receptor modulators (SERMs) are synthetic non-steroidal drugs that act as both ER agonists and ER antagonists [39,40]. Common selective estrogen receptor modulators are tamoxifen, raloxifene, and clomiphene citrate (CC). By contrast, fulvestrant is a selective estrogen receptor downregulator (SERD) that results in ER degradation or downregulation and blocks the proliferation of breast cancer cells [41]. Apart from this, the non-steroidal ligand G-1 (1-[4-(6-bromobenzo[1,3] dioxol-5yl)-3a,4,5,9b-tetrahydro-3H-cyclopenta[c]quinolin-8-yl]-ethanone), which acts as a selective agonist for GPER, has been shown to induce the expression of genes by activation of GPER rather than the classical ERα or ERβ (as shown in Figure 2).

## 3. Physiological and Pathological Function of ERs

It is obvious that some diseases are associated with the levels of estrogen and estrogen receptors. ERs play crucial roles in breast carcinogenesis, with ERα-positive cancer represents approximately 75% of all breast cancer patients, while the presence of estrogen can promote the growth of tumors [42]. There are two kinds of estrogen receptors, ERα and ERβ, in ovarian tumors. ERα is increased and ERβ is decreased in malignant tumors compared with corresponding benign tumors. GPER is also involved in the proliferation of ovarian cancer cells, while GPER mRNA, as well as GPER protein, is present in both primary and malignant ovarian tumor tissues. In general, estrogen stimulation of ovarian cancer cell proliferation requires both GPER- and ER-mediated complete epidermal growth factor receptor (EGFR) signaling [43]. On the other hand, endometrial cancer is also sensitive to estrogen. The long-term administration of estrogen alone can cause endometrial cancer in postmenopausal women [44].

The cyclic secretion of estrogen and its interaction with related receptors plays a prominent role in ovarian function and the regulation of endometrial proliferation and differentiation. If hormone levels are consistently disturbed in females, this affects ovarian function, which leads to the formation of cysts in the ovary. Similarly, the endocrine and metabolic abnormalities related to PCOS can increase the risk of endometrial hyperplasia and cancer in women [45]. Thus, impaired fertility in patients with PCOS may result not only from anovulation, but also from endometrial dysfunction.

Estrogen actions are mediated via genomic pathways by ERα and ERβ. Upon the binding of estradiol to ERα or ERβ in the cytoplasm, a conformational change occurs, inducing dimerization of the receptor. This complex is then translocated to the nucleus, where it binds to chromatin at the ERE sequences and alters the transcription of target genes by recruiting transcription factors. Apart from this, it is widely known that more rapid non-genomic pathways (also known as membrane effects) exist. Rapid responses to estrogen involve the mobilization of various second messengers, such as cyclic adenosine monophosphate (cAMP) and calcium ions (Ca2+), or the activation of intracellular kinase pathways (as shown in Figure 2). These observations show that GPER can mediate these fast effects to activate various signal transduction cascades, such as mitogen-activated protein kinase (MAPK), protein kinase C, and phosphatidylinositol 3-kinase (PI3K). Furthermore, E2 binds ERα and ERβ localized in the plasma membrane to affect cellular signaling through rapid membrane initiation events, which is also part of non-genomic signaling. Indeed, in addition to genomic and non-genomic ligand-dependent estrogen signaling, ERs can also be activated in the absence of E2 or another suitable ligand. This is attributed to the phosphorylation of the receptors on some residues or their associated coregulators.

Some genetically engineered or inherited animal models have shown symptoms of PCOS, which are due to inappropriate hormone exposure or ER changes affecting endocrine homeostasis, leading to the development of ovarian abnormalities and a reduction in fertility [46,47]. Paying attention to animal PCOS models is helpful in order for us to better understand the disease characteristics of PCOS (as shown in Table 1).

## 4. Functions of ERs in PCOS

### 4.1. ERs with Follicular Formation/Ovulation

Although it is generally believed that disorders of the hypothalamic–pituitary–ovary axis are a significant cause of cystic formation, the cystic follicle maintains a static condition without degeneration after ovulation failure, which is considered another reason for the development of cysts [54]. In mice, abnormal ERβ expression in the ovaries results in the failure of dominant follicles to develop consistently [55]. Ovarian estrogens are thought to regulate follicular maturation locally in the ovaries and to stimulate the proliferation of GC during dominant follicle growth. Recently, the relationship between GPER and oocyte maturation was reported. The research study showed that the maturation of carp oocytes significantly decreased when they were incubated with either E2 or GPER agonist G-1 [56]. Another study showed that in cumulus granulosa cells from patients with PCOS, low levels of E2, accompanied by high levels of GPER, might inhibit human oocyte maturation [57]. Meanwhile, GPER small interfering RNA knockdown mice indicated that GPER had a stronger inhibitory action on primordial follicles, indicating that GPERs might play a crucial part in oocyte maturation [58]. Estrogens inhibit meiotic maturation of full-grown oocytes by activating the estrogen receptor GPER.

Estrogen has been reported to affect some ovarian functions via autocrine or paracrine actions, most significantly enhancing the effect of FSH on granulosa cells [59]. ERs are expressed in GC and theca cells (TC) in developing follicles. Based on these considerations, in conditions characterized by ovulation dysfunction, such as PCOS, altered ovarian ER expression may have an essential role. Mice with the ERα gene disrupted developed a phenotype similar to PCOS, with high circulating LH concentrations and ovaries characterized by multiple hemorrhagic and cystic follicles with non-ovulation [60,61]. Likewise, by detecting mRNA and protein levels of ERα and ERβ in GC and TC from regularly circulating ovaries and polycystic human ovaries, we observed that the expression of ERα in GC was higher than in TC, but ERβ was expressed similarly in both cell types [62]. It seems that ERα has a significant advantage compared to ERβ in poor follicular development and ovulation failure in ovary syndrome. Rumi et al. used zinc finger nuclease (ZFN)-mediated genome editing to generate ER knockout rats, in which they could observe the phenomena of uterine dysplasia, polycystic ovaries, and ovulation defects [63]. Specifically, these animals failed to express functional ERα proteins and showed obvious abnormalities in postnatal growth, fertility, female genital tract development, and response to E2. In humans, an 18-year-old woman was reported to have a homozygous ESR1 mutation in a completely conserved residue, with an ovarian cyst and a small uterus despite having elevated circulating serum E2 [64]. Interestingly, ERβ knockout mice also showed morphological characteristics of abnormal follicular development, as well as decreased ovulation ability [65]. Compared with wild-type mice, they had earlier atretic follicles and fewer corpora lutea, although ER gene knockout mice can reproduce [66,67]. Similarly, another experiment also showed female ERβ knockout mice to have poor fertility, characterized by lower ovulation numbers, fewer pregnancies, and preovulatory follicles exhibiting a weak response to FSH-induced differentiation [68]. Thus, abnormal expression of ERs (ERα and ERβ) may be related to poor oocyte development and ovulatory failure in PCOS patients.

### 4.2. ER Changes Associated with Endometrium

During normal menstrual cycles, the endometrium undergoes rapid cycling, proliferation, and growth in response to estrogen through the action of specific steroid receptors ERα and the ERβ [69]. During the follicular phase, E2 induces the rapid growth of the uterine endometrium and increases endometrial sensitivity to estrogen by increasing ERα levels. Conversely, at the luteal phase of the menstrual cycle, the corpus luteum continuously secretes progesterone and reduces estrogen to provide an appropriate uterine environment for maintaining the pregnancy. Thus, improper estrogen action affecting maximal uterine acceptance capability may change the normal expression of genes and reduce fertility in women with PCOS or increase the rate of spontaneous miscarriage.

ERα and the ERβ have different cellular localizations in the human endometrium. In general, ER (ERα and ERβ) expression reaches a maximum at the late proliferative stage and decreases at the secretory phase of the menstrual cycle [70,71]. For example, ERα mRNA is expressed in both the endometrial epithelial and stromal cells during the menstrual cycle, whereas ERβ mRNA is found predominantly in glandular epithelial cells [72]. In addition, the expression level of ERα mRNA in the uterus is more prominent than ERβ mRNA. GPER is localized in the plasma membrane and in the endoplasmic reticulum, which is considered to regulate the growth and proliferation of endometrial cells through its interaction with ERα [73,74,75].

There is growing clinical and experimental evidence showing that ER is an endometrial marker in patients with PCOS. Estrogen-induced uterine hyperplasia occurs by binding uterine epithelial ERα in adult mice to actively inhibit epithelial apoptosis in the uterus [76]. The same scenario is observed for ovulatory PCOS, where high levels of ERα expression are observed. In women with PCOS, ERα expression in the endometrium continues to enter the secretion stage, showing high expression in the stroma and luminal epithelium [77]. One woman with a homozygous ERα mutation and rats lacking ERα showed similar phenotypes and infertility similar to what has been observed in patients with PCOS [61,64].

**Figure 3:**
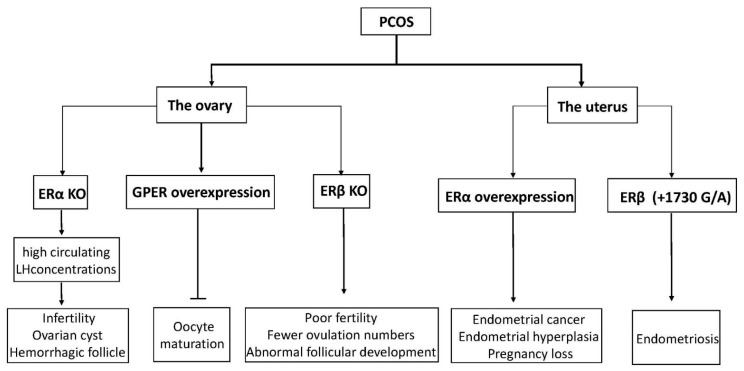
A diagram of physiological phenomena to explain disordered expression in the ER family. Arrows indicate direct effects, while no arrows indicate inhibition.

In women with ovulatory dysfunction, the endometrium, as a target tissue for estrogen, is prone to hyperplasia and cancer [78,79,80]. PCOS symptoms are significantly related to endometrial cancer risk. Women under age 50 with PCOS have four times higher incidence of endometrial cancer than women without PCOS [81]. Based on previous research, increased ERα expression in the endometrium of PCOS women at both gene and protein expression levels has been observed, suggesting that the endometrium is more sensitive to estrogen, possibly explaining the significant increased incidence of hyperplasia and endometrial cancer, as well as the decreased ability to continue pregnancy [82,83]. Moreover, an ERβ polymorphism (+1730 G/A) was associated with the development of susceptibility to PCOS in humans [84]. Polymorphisms in identified ER-α were found to have an association with the development of endometrial cancer [85]. Furthermore, changes in ERα expression levels and the ERα/ERβ ratio in PCOS patients were higher than in the normal group, indicating outstanding ERα-mediated actions in the endometrium, which may be related to endometrial hyperplasia and endometrial cancer [86]. Previous studies have shown that p160 steroid receptor coactivator is increased in the secretory-phase endometria of women with PCOS, which further increases endometrial ERα expression to stimulate endometrial proliferation [86,87]. Abnormal estrogenic environments may alter endometrial receptivity in women, resulting in blastocyst implantation failure or a malformed abortion after implantation (the functions of ERs in PCOS are shown in Figure 3).

## 5. Selective Estrogen Receptor Modulators

For women with PCOS, the mainstream method of treatment for anovulatory infertility is to restore mono-ovulation. The mechanisms by which modulators control the activity of ERs remain a subject of ongoing research. SERMs exert either agonistic or antagonistic biological properties, depending on different tissues. They are designed to compete with estrogen by binding ERs and modulating ER activity by changing the cofactors associated with ERs [88]. Each compound has different tissue specificity.

Clomiphene citrate is an oral SERM consisting of two isomers, zuclomiphene and enclomiphene, which compete for receptor binding sites with endogenous estrogens. The former has a strong biological activity to induce ovulation and has a much longer half-life than enclomiphene, being detected in plasma 1 month after administration. Its mechanism of action is to inhibit negative estrogen feedback to the hypothalamus and pituitary gland by blocking ERs. Therefore, an increased secretion of gonadotropins from the anterior pituitary stimulates follicular development and ovulation [89]. The method of CC administration is 50–150 mg daily for 5 days, beginning on day 3–5 after spontaneous or progestin-induced withdrawal bleeding [90]. For women with PCOS, ovulation time in the stair–step clomiphene group was reduced by an average of 30 days and increased ovulation rates were observed compared to those in traditional protocols [91]. Therefore, the stair–step protocol has been shown to quickly increase the probability of ovulation.

Clomiphene citrate is related to an increased risk of multiple pregnancies, estimated at 10%, whereas hyperstimulation syndrome is rare [92]. Some with PCOS have a strong response to CC even at the lowest dose; for example, experimental evidence suggests that 14% of women with low doses of CC develop three or more follicles [93]. In addition, the anti-estrogenic effect of CC may cause proliferation of the endometrium, however the importance of this relative to the chances of conception is unclear [94]. Other side effects are common but transient, such as hot flashes, headaches, mood changes, and blurred vision [95]. Although mild ovarian enlargement is relatively common, side effects of CC use resulting in a full blown ovarian hyperstimulation syndrome (OHSS) have been rare in nearly 40 years of practice [96]. Occasional cyst formation may be treated conservatively. In spite of this, CC remains the first-line therapy for ovulation induction in women with PCOS. The drug is low-cost and oral administration is patient-friendly. Adverse reactions are relatively rare, and there is a large amount of clinical data on the safety of the drug.

Tamoxifen is a first-generation estrogen receptor modulator and is chemically very similar to CC. It manifests as an ER antagonist in breast tissue and has estrogen-like effects in bone tissue, the cardiovascular system, and uterine cells [97]. Although its main role is as an adjuvant treatment for breast cancer, it is also used to stimulate ovulation. Its ovulation and pregnancy rates seem to be comparable to CC [98]. As a replacement for CC, tamoxifen is administered at 20–40 mg in the same manner [99].

Raloxifene is the second generation of SERM, which is different from the first generation in terms of its chemical structure; it has similar anti-estrogen effects, however it causes a very low degree of stimulation of the endometrium [100]. In human uterine cell lines, synthetic anti-estrogens, including tamoxifen and raloxifene, activate the ER-dependent AP-1 site. When raloxifene binds to ER receptors, this flexible orientation of the basic side chain leads to a conformational change of the ER, thus significantly blocking the receptor-mediated pathway [101].

The use of aromatase inhibitors (AIs) to treat anovulatory cycles is a new approach. AIs (such as anastrozole and letrozole) inhibit the conversion of androstenedione and testosterone into estrogens by inhibiting aromatase [102,103]. Letrozole has no significant effect on endometrial receptivity and is the most commonly used aromatase inhibitor to induce ovulation. It is utilized at a daily dose of 2.5–7.5 mg for five days, from the third day of the menstrual cycle [104]. Proponents believe that aromatase inhibitors are superior to CC, easy to use, and safe, but clinical consequences show a risk of osteoporosis, as well as adverse effects on the cardiovascular system and lipid levels [105,106]. Although some aromatase inhibitors may replace CC as first-line drug therapies, CC is still considered the drug of first choice for women with PCOS.

SERM treatment is a viable, available adjunctive option for infertility in PCOS patients, with CC being the most studied and efficient method. In addition, the levels of androgen in the GCs of PCOS patients can be reduced to improve oocyte quality [107]. Further, we should emphasize that unhealthy living conditions affect estrogen levels. Changes in lifestyle and diet can delay or prevent the pathological effects of estrogens and contribute to expressing the right type of ERs in the right tissues.

## 6. Concluding Remarks

In premenopausal women, the ovaries produce the major estrogen 17β-oestradiol, which circulates to target organs and cells. Estrogen regulates a large amount of biological and physiological processes through estrogen receptors. Its mechanisms are complex and varied, mainly involving the direct binding of ERα and ERβ to specific DNA sequences or activating intracellular cascades that result in non-genomic control of transcription. Research into such mechanisms can help in the design of therapeutic strategies for diseases involving estrogen receptors, such as PCOS. The physiological effects of ER-mediated estrogen remain elusive. The identification of the ER is considered to be an effective target for the treatment of PCOS and the use of SERM drugs contributes to female health. However, the use of therapies targeting ERs in treating diseases carries a risk of undesirable effects. Most importantly, further research is needed to develop new molecules that modulate ERs in selective metabolic tissues to prevent and treat diseases affected by estrogens.

## Figures and Tables

**Table 1:** The symptoms of polycystic ovary syndrome (PCOS) caused by changes in the endocrine systems of animal models.

Classification of Organisms	Year	Species	Type	Estrous Cycle	Ovarian Cyst	Fertility	Notes	Reference
Rodent models	2009	Mouse	ER α KO	Irregular	Yes	Lost (age of 6 months)	The formation of hemorrhagic follicle; androgen level (↑)	[48]
	1986	Rat	Treated with estradiol-valerate	Cease	Yes	NA	Anovulation; ovarian weights declined significantly	[49]
	2004	Mouse	ERβ KO (elevated LH)	NA	No	NA	Increased steroidogenic enzyme expression; androgen level (↓)	[50]
Nonhuman primate models	2008	Cynomolgus monkey	Spontaneous PCOS	A prolonged menstrual cycle length (up to 161 days)	Yes	NA	Endometrial hyperplasia	[51]
	2002	Female Rhesus Monkeys	Exposed prenatally to androgen	Irregular	Yes	NA	Delayed menarche; androgen level (↑)	[52]
Other models	2001	Female sheep	Exposed prenatally to androgen	Irregular	Yes	NA	Increased ovarian volume; anovulation	[53]

Note: ↑ = upregulated; ↓ = downregulated; NA: not available or not assessed.

## Data Availability

Not applicable.
